# Targeted Tuberculosis Surveillance Testing in Patients With Inflammatory Bowel Disease: Is That the Best Way Forward?

**DOI:** 10.1093/crocol/otab027

**Published:** 2021-06-11

**Authors:** Kofi Clarke

**Affiliations:** Penn State College of Medicine, Milton S. Hershey Medical Center, Hershey, PA, USA

A well-defined strategy of early identification, treatment, contact tracing with evaluation, and appropriate treatment of exposed individuals limits secondary tuberculosis (TB) transmission.^1^

The overall incidence of TB has trended downward in the United States; however, the pace of decline has not been linear. Between 2012 and 2017, there was an average annual decline of 2.2% compared to a much more impressive rate of 6.7% between 2007 and 2012.^2, 3^

Reports of cases of TB from Health Departments in the 50 US states and the District of Columbia include standard information on demographics, clinical features, and predisposing or risk factors for infection.

TB exposure or infection often results in the development of latent infection (LTB). The overall case rate in the United States is estimated to be 2.7 cases per 100,000 persons with almost half of the cases reported from New York, Texas, California, and Florida. In immunocompetent persons, there is a 4%–6% lifetime risk of progression of LTB and a higher likelihood of progression in immunosuppressed individuals.^4–7^

More concerning is the estimated 14-fold increased risk of TB reactivation in patients treated with biologic therapies.^8, 9^

The standard of care for patients with inflammatory bowel disease (IBD) on biologic therapy includes pretreatment evaluation for LTB and annual testing during therapy. This is primarily done to reduce the risk of reactivation and new infections in this high-risk patient group. The annual retesting is also a prerequisite for continued insurance coverage for biologics and small molecule therapy.

Modalities for testing in clinical practice are primarily tuberculin skin tests and interferon-gamma release assays (IgGRA) which include QuantiFERON gold testing.

Previous studies have evaluated factors that may affect the performance of these tests and also assessed their performance in the same individuals. Other studies have evaluated how the tests compare to each other and conversion rates while on therapy. Several of these studies were done in other parts of the world where the epidemiology of TB is different than in the United States and in cohorts of patients with other immune-mediated diseases and not IBD specifically.^10–14^

In this issue of *Crohn’s & Colitis 360*, Fine et al^15^ report the findings of their study on a cohort of patients in Rhode Island. They conclude that patients on biologic therapy “unnecessarily undergo surveillance testing for TB.” In addition, they observed that “patients with IBD on biologic therapy are screened annually for TB at a higher rate compared to non-IBD patients.” They infer that patients with IBD on biologic therapy in low-incidence regions should not undergo yearly testing unless they are considered high risk or have had a potential exposure. Part of their recommendation is based on the overall high cost of care/patient with IBD and the need for cost containment.

We need to see this data replicated from several parts of the United States especially in the IBD patient cohort at the highest risk of reactivation of LTB and in the areas with the highest case rates.

The estimated cost of IgGRA testing for TB is estimated to be $50.00, which pales in comparison to the devastating effects and potential loss of life from disseminated TB in a patient on biologic therapy.

Targeted TB screening and surveillance of patients with IBD on biologics sound reasonable in principle. This should include a detailed history with specific emphasis on risk factor identification and a detailed social and travel history within the preceding year.

A few lingering questions and concerns that should give us pause?

1. This was a retrospective study in an area of low incidence and prevalence of TB—can we extrapolate the results?
2. Philosophically, efforts aimed at cost containment are essential to continue to provide state-of-the-art care; but should we be focusing on the low $ items or the higher $$$$ contributors to cost?
3. Should the reassuring data from lower TB screening and surveillance rates in patients with other immune-based conditions be a source of comfort?

The work by Fine et al^15^ on this issue is a call to revisit this issue in a more considered manner. Prospective studies on the performance of available testing with targeted testing in the highest risk cohort in the United States should be a key aspect of the rethink.
